# Melanoma Affects the Composition of Blood Cell-Derived Extracellular Vesicles

**DOI:** 10.3389/fimmu.2016.00282

**Published:** 2016-07-26

**Authors:** Nina Koliha, Ute Heider, Tobias Ozimkowski, Martin Wiemann, Andreas Bosio, Stefan Wild

**Affiliations:** ^1^R&D Reagents, Miltenyi Biotec GmbH, Bergisch Gladbach, Germany; ^2^Institute for Lung Health, IBE R&D gGmbH, Münster, Germany

**Keywords:** exosome, nanoparticle tracking analysis, flow cytometry, platelets, B cells, moDC

## Abstract

Extracellular vesicles (EVs) are specifically loaded with nucleic acids, lipids, and proteins from their parental cell. Therefore, the constitution of EVs reflects the type and status of the originating cell and EVs in melanoma patient’s plasma could be indicative for the tumor. Likewise, EVs might influence tumor progression by regulating immune responses. We performed a broad protein characterization of EVs from plasma of melanoma patients and healthy donors as well as from T cells, B cells, natural killer (NK) cells, monocytes, monocyte-derived dendritic cells (moDCs), and platelets using a multiplex bead-based platform. Using this method, we succeeded in analyzing 58 proteins that were differentially displayed on EVs. Hierarchical clustering of protein intensity patterns grouped EVs according to their originating cell type. The analysis of EVs from stimulated B cells and moDCs revealed the transfer of surface proteins to vesicles depending on the cell status. The protein profiles of plasma vesicles resembled the protein profiles of EVs from platelets, antigen-presenting cells and NK cells as shown by platelet markers, co-stimulatory proteins, and a NK cell subpopulation marker. In comparison to healthy plasma vesicles, melanoma plasma vesicles showed altered signals for platelet markers, indicating a changed vesicle secretion or protein loading of EVs by platelets and a lower CD8 signal that might be associated with a diminished activity of NK cells or T cells. As we hardly detected melanoma-derived vesicles in patient’s plasma, we concluded that blood cells induced the observed differences. In summary, our results question a direct effect of melanoma cells on the composition of EVs in melanoma plasma, but rather argue for an indirect influence of melanoma cells on the vesicle secretion or vesicle protein loading by blood cells.

## Introduction

Exosomes are extracellular vesicles (EVs) that are formed by inward-budding of late endosomes that fuse with the plasma membrane to release exosomes. Therefore, exosomes exhibit the same membrane orientation as cells by displaying membrane bound proteins on their surface (1, 2). Exosomal proteins are transferred from complexes involved in exosome biogenesis, such as the tetraspanin CD63 (3) or from the originating cell membrane (2). Tetraspanin proteins are, therefore, used as exosome markers (4, 5) and cell-type-specific markers on exosomes are thought to indicate the originating cell type (6).

In melanoma patients, the amount of plasma exosomes was reported to be increased in comparison to healthy donors (7). This raises the question whether the surplus of exosomes derives from the tumor itself or from blood cells. Melanoma-derived exosomes can prevent anti-tumor immune responses by promoting the generation of an immunosuppressive cell subset (8) and are able to promote tumor progression (9). Tumor-derived exosomes were found to be immunosuppressive as well as immune activating. On the one hand, tumor exosomes inhibit the activity of natural killer (NK) cells and cytotoxic T cells (10, 11) or induce apoptosis of T cells by galectin-9 or Fas ligand (12–14). On the other hand, they are able to activate cytotoxic T cells via tumor antigens after being loaded onto dendritic cells (15, 16).

Since Caby and colleagues found exosomes in human blood, platelets are considered as major contributors to the EV pool found in plasma (17). Major histocompatibility complex (MHC) class II and CD86 on plasma exosomes point at antigen-presenting cells (APCs) as exosome source (17, 18). Additionally, activated cells have to be taken into account as cell stimulation alters the protein loading or secretion of exosomes from B cells (19) and T cells (20, 21), respectively. The contribution of different cell types to the pool of plasma EVs is considered important because blood cell-derived EVs are known to modulate immune responses and tumor progression in various ways [for a review, see Ref. (1, 22)].

Uncovering the parental cells of plasma EVs might give insight into the intercellular communication between melanoma cells and immune cells. This requires that plasma EVs actually carry cell-type-specific markers enabling the identification of their parental cells. So far, most of the studies focused on exosomes released by a distinct cell type and differed in the applied methods for protein detection. For example, platelet EVs were characterized by electron microscopy (23) and mass spectrometry (24), while ELISA and flow cytometry were applied to investigate EVs from B cells (19, 25–28) and monocyte-derived dendritic cells (moDCs) (29–31). To achieve an extensive protein profiling of EVs from different sources, we isolated EVs from plasma of melanoma patients and healthy donors as well as from diverse blood cell types and analyzed them by a multiplex bead-based platform (32). We identified surface proteins on EVs that were adopted from parental cells and allowed to distinguish EV types secreted from different cell types.

Interestingly, from three markers, which were suggested as melanoma markers (33–35), only CD49e was detected in five out of eight melanoma plasma samples, whereas MSCP and CD146 were hardly detectable on plasma EVs isolated from melanoma patients. This indicates that only a minor portion of plasma vesicles derive from melanoma cells. Still, we found that the signal intensities for five surface proteins differed between plasma EVs from healthy donors and melanoma patients. We suggest that tumor cells induce a change of the vesicle composition in plasma of melanoma patients by altering the EV secretion or EV protein loading of blood cells.

## Materials and Methods

### Cell Isolation, Culture, and Stimulation for Vesicle Production

Melanoma cell lines were grown in RPMI1640 (biowest, L0501-500) with 10% FCS (Biochrom, S 0415), 2 mM l-glutamine (Lonza, BE-17-605E), 50 U/ml penicillin, and 50 μg/ml streptomycin (Thermo Scientific, SV30010). For EV production, the cells were washed with phosphate buffered saline (PBS) and cultured for 72 h in FCS-free medium to avoid any contamination with bovine EVs. The percentage of apoptotic cells (AnnexinV^+^, Miltenyi Biotec, 130-093-060) was below 5%.

The donors of whole blood, buffy coats, and leukapheresis that were used for the following cell isolations gave written informed consent in accordance with the Declaration of Helsinki.

Platelets were isolated from fresh whole blood that was diluted with an equal volume of Krebs Ringer buffer (100 mM NaCl, Th Geyer 1367, 4 mM KCl, AppliChem A1428, 20 mM NaHCO_3_, Merck 1063291000, 2 mM Na_2_SO_4_, Sigma Aldrich 403008-100G, 4.7 mM citric acid, VWR EM-CX1723-1, 14.2 mM tri-sodium citrate, Merck 1.06448.1000) at pH 7.4 and centrifuged at 190 × *g* for 10 min (23). To deplete leukocytes and erythrocytes the platelet-rich plasma was centrifuged at 100 × *g* for 20 min. Platelets were pelleted at 1,000 × *g* for 15 min and washed twice with Krebs Ringer buffer. 1 to 9 × 10^7^ platelets per milliliter whole blood were isolated and platelet purities ranged from 82 to 99%. After adjusting to 1 × 10^9^ platelets per milliliter, they were activated with 50 nM Calcium Ionophore (Sigma Aldrich, C7522-1MG) and 10 mM calcium chloride (Sigma Aldrich, C3306-100G) for 30 min at room temperature (36).

T cells were isolated from Buffy Coats by Pan T Cell Isolation Kit (Miltenyi Biotec, 130-096-535) with purities of 96–99%. To generate as many EVs as possible the protocol by van der Vlist et al. was used with minor modifications (21). Briefly, cells were cultured in TexMACS medium (Miltenyi Biotec, 130-097-196) without serum with 5 U/ml IL-2 (Miltenyi Biotec, 130-097-743) and with 2.5 μg/ml CD28 (clone 15E8, Miltenyi Biotec Cat# 130-093-375 Lot# RRID:AB_1036134) in CD3 (clone OKT3, Miltenyi Biotec Cat# 130-093-387 Lot# RRID:AB_1036144) coated tissue culture flasks for 24 h with viability rates >90%. After activation, 75–95% of T cells were positive for the T cell activation marker CD69 (Miltenyi Biotec Cat# 130-092-160 Lot# RRID:AB_615102).

Natural killer cells were isolated from buffy coats using the MACSxpress^®^ NK Cell Isolation Kit and cultured in TexMACS GMP medium (Miltenyi Biotec, 170-076-309) with 5% human AB serum (Life Technologies, 34005100) and 500 U/ml Proleukin S (Novartis, 2238131) for 14 days.

Monocytes were isolated from Buffy coats after Ficoll gradient by immunomagnetic cell sorting using CD14 MicroBeads (Miltenyi Biotec, 130-050-201) with purities of 92–98% and cultured in RPMI1640 (biowest, L0501-500) with 2 mM l-glutamine (Lonza, BE-17-605E), 50 U/ml Penicillin, and 50 μg/ml Streptomycin (Thermo Scientific, SV30010) for 24 h with viability rates >90%.

To generate moDCs, monocytes were isolated from leukapheresis by immunomagnetic cell sorting using CliniMACS CD14 Beads (Miltenyi Biotec, 272-01) and the CliniMACS Prodigy^®^ system (Miltenyi Biotec, Germany). 2 to 6 × 10^6^ monocytes per milliliter were cultured in RPMI (Lonza, BE12-167F) with 2 mM l-glutamine (Lonza, BE-17-605E), 1% autologous serum, 250 IU/ml IL-4 (Miltenyi Biotec, 170-076-135), and 800 IU/ml GM-CSF (Miltenyi Biotec, 170-076-112). After 2 and 4 days, half of the medium was replaced by fresh medium adjusted to the same final cytokine concentrations. On day 6, half of the medium was replaced by fresh medium to reach final concentrations of 1 μg/ml PGE_2_ (Merck, 538904-1MG), 1000 IU/ml TNF-α (Miltenyi Biotec, 170-076-103), 1000 IU/ml IL-6 (Miltenyi Biotec, 170-076-104), and 200 IU/ml IL-1ß (Miltenyi Biotec, 170-076-102). To isolate EVs, supernatants of immature moDCs were harvested on day 2, 4, and 6, and supernatants from mature moDCs on day 7 and 10.

B cells were isolated from Buffy coats after Ficoll gradient by immunomagnetic cell sorting using CD19 MicroBeads (Miltenyi Biotec, 130-050-301) with purities of 97–99%. 2 × 10^6^ B cells per milliliter were cultured in StemMACS HSC Expansion Media XF (Miltenyi Biotec, 130-100-473) with 5% EV-depleted human AB serum (Gemini, 100-512). To stimulate the cells, 1 μg/ml CD40-Ligand, cross-linking antibody (Miltenyi Biotec, 130-098-776), and 20 IU/ml IL-4 (Miltenyi Biotec, 130-093-919) were incubated for 30 min at room temperature before cells were added to the medium for 4 days. On the day of EV harvest, the viability rates were >90%. Eighty-five to 93% of activated B cells were positive for CD80 (Miltenyi Biotec Cat# 130-101-218 Lot# RRID:AB_2571526) and 83–95% were positive for CD86 (Miltenyi Biotec Cat# 130-100-100 Lot# RRID:AB_2571527).

### Blood Samples

Blood was obtained from healthy volunteers after written informed consent on nine different days with the safety blood collection set 21G x 3/4″ (Greiner, 450085) in vacutainers with 0.105 M sodium citrate (BD, 366575) or 17 IU/ml heparin (BD, 367526) as anticoagulant. For the isolation of platelets and plasma EVs, blood was anticoagulated with sodium citrate and donors had not consumed alcohol or ibuprofen in the last 24 h and did not use aspirin, heparin, or anti-histamines in the preceding 2 weeks to exclude impacts of these drugs on platelet activation and exosome secretion. Donors were fasting to decrease lipoproteins that might sediment with EVs during ultracentrifugation and contaminate the sample. Blood was drawn at the same time of day (i.e., between 08:30 and 09:30 a.m.) to minimize circadian rhythm effects. The first 3 ml of blood were discarded to reduce contamination with activated platelets and fibroblasts. Donors were not pregnant. Blood was centrifuged 1 h after blood drawn at the latest.

After declaration of informed consent as approved by the Ethics Committee of the *Friedrich-Alexander Universität Erlangen Nürnberg (Ethik-Kommission/Re.-No. 4602)* blood from melanoma patients was collected with heparin as anticoagulant. Plasma was isolated and stored at −80°C until use.

Age, sex, smoking habit, and medication of the healthy donors and the melanoma patients are summarized in Table S2 in Supplementaray Material.

### Isolation of Extracellular Vesicles

Cell culture supernatants were depleted from cells and large cell debris by centrifugation at 2,000 × *g* for 30 min. Dead cells and cell debris were depleted by centrifugation at 10,000 × *g* for 45 min and larger vesicles by filtration through 0.22 μm membranes. EVs were pelleted at 108,000 × *g* for 2 h in a Beckman Avanti J30i centrifuge with an appropriate rotor (JA-30.50 Ti) and washed with PBS. To isolate plasma EVs whole blood was centrifuged at 1,000 × *g* for 10 min. Plasma was diluted with an equal volume PBS before performing the centrifugation steps described above. Plasma EVs were filtrated through a 0.22 μm membrane in between the two ultracentrifugation steps at 108,000 × *g* for 2 h.

Extracellular vesicle pellets were resuspended in a minimal volume of PBS. The EV concentration was measured indirectly by BCA Protein Assay (Pierce, 23227) using BSA as standard.

### Nanoparticle Tracking Analysis

Size distribution and concentration of EVs were measured with a Nano Sight LM10 instrument equipped with a 532 nm laser and an EMCCD camera (Malvern Instruments Ltd., UK). For data analysis, NTA software version 3.1 was used. All measurements were carried out with optimized settings from the same experienced operator to achieve comparable results. EVs from different sources were serially diluted in essentially particle-free PBS. Concentration was calculated from the particle concentration step ideally suited for tracking analysis (ca. 5 × 10^8^ particles/ml) and the respective dilution factor. The value obtained this way together with the protein concentration of the diluted sample was used to calculate the protein amount per particle.

### Scanning Electron Microscopy

Morphology and size of EVs were analyzed by scanning electron microscopy as described by Lui (37). Ten microgram primary melanoma EVs were diluted in 0.22 μm filtered PBS for a final volume of 50 μl and loaded on a silicon carrier. EVs were washed three times with 0.22 μm filtered PBS and fixed in 2% paraformaldehyde (Miltenyi Biotec, 130-090-477) for 10 min and washed again. Subsequently, EVs were fixed in 2.5% glutaraldehyde (Alfa Aesar, A17876) for 10 min and washed five times with distilled water. Before imaging, a MED010 sputter was used to sputter coat the sample with gold–palladium for 90 s. A Zeiss Supra 55 Scanning Electron Microscope (Zeiss, Germany) was used at a voltage of 2 kV. The diameter of 100 EVs was measured to determine the average size by using the software Image J.

### Dynamic Light Scattering

Dynamic light scattering was used in addition to NTA and scanning electron microscopy for EV sizing. 1.5 μg EVs from a primary melanoma cell line or 10 μg plasma EVs were diluted in 500 μl distilled water and measured at the Delsa nano HC (Beckman Coulter). To determine the EV size, the average of three independent measurements was calculated.

### Multiplex Bead-Based Platform

Polystyrene beads were labeled with two dyes in varying amounts to distinguish 39 different bead populations by flow cytometry in the FITC and PE channel (32). Each bead population was coupled with a different capture antibody (Table S3 in Supplementary Material).

Approximately 800 beads per bead population were incubated with isolated EVs or cell culture supernatant at 4°C overnight in 100 or 300 μl, respectively. To adjust the volume of different samples PAP buffer was used consisting of PBS with 0.1% Pluronic (Gibco, 24040-032) and 0.09% azide (VWR, 1.06688.0250). To remove unbound EVs, the beads were washed in PAP and pelleted at 3,000 × *g* for 5 min. The beads were resuspended in 100 μl PAP and bound EVs were stained with a cocktail of 0.5 μg each of APC-conjugated anti-CD9 (clone SN4, Miltenyi Biotec Cat# 130-103-956 Lot# RRID:AB_2571528), anti-CD63 (clone H5C6, Miltenyi Biotec Cat# 130-100-182 Lot# RRID:AB_2571529), and anti-CD81 (clone JS-81, BD Biosciences Cat# 551112 Lot# RRID:AB_398491) antibodies.

### Flow Cytometry Analysis

For analysis, the flow cytometer MACSQuant Analyzer 10 with the corresponding software (Miltenyi Biotec) was used.

For cell analysis, 5 × 10^5^ monocytes and 5 × 10^5^ moDCs were each stained with CD14-FITC (Miltenyi Biotec Cat# 130-080-701 Lot# RRID:AB_244303), CD40-APC (Miltenyi Biotec Cat# 130-094-137 Lot# RRID:AB_10828346), and CD86-PE (Miltenyi Biotec Cat# 130-094-877 Lot# RRID:AB_10839702) or with CD209-APC (Miltenyi Biotec Cat# 130-092-871 Lot# RRID:AB_871640). Stainings were performed in a total volume of 110 μl with final antibody dilutions of 1:11 with FcR Blocking reagent (Miltenyi Biotec) in PEB (PBS with 0.5% BSA and 2 mM EDTA) for 10 min at 4°C. After washing with PEB, cells were resuspended in 100 μl PEB, dead cells were stained with propidium iodide and the sample was analyzed by MACSQuant Analyzer 10 (Miltenyi Biotec). At least 10,000 events were measured per sample. In the forward/side scatter, debris was excluded from the analysis. Dead cells were identified by staining with propidium iodide and excluded from the analysis. Gates were set considering the stainings with isotype control antibodies. For evaluation, MACSQuantify software version 2.6 (Miltenyi Biotec) was used.

For the multiplex platform, appropriate settings were selected as previously described (32). The signals depicted in the figures are background corrected signals or normalized signals. Background corrected signals were calculated for each bead type by subtracting the signal obtained with beads and the staining cocktail from the signal of captured and stained EVs. For the NK cell derived EVs isolated from medium with human AB serum, an additional background control was used: the protein amount of an equivalent volume of medium, including human AB serum without exposure to cells was used and the signals were subtracted from the EV sample signals. Signals were normalized to the mean signal intensity obtained with the anti-CD9, CD63, and CD81 beads.

### Statistics

To create the heat map depicted in Figure 3, the public software Multi Experiment Viewer (MeV) was applied (38). For unsupervised hierarchical clustering, average linkage clustering was performed with the distance metric approach using Pearson correlation, including optimization of sample leaf order.

*T*-test calculations were performed using Microsoft Excel.

## Results

### Size and Concentration of Isolated Extracellular Vesicles

**Figure 1:**
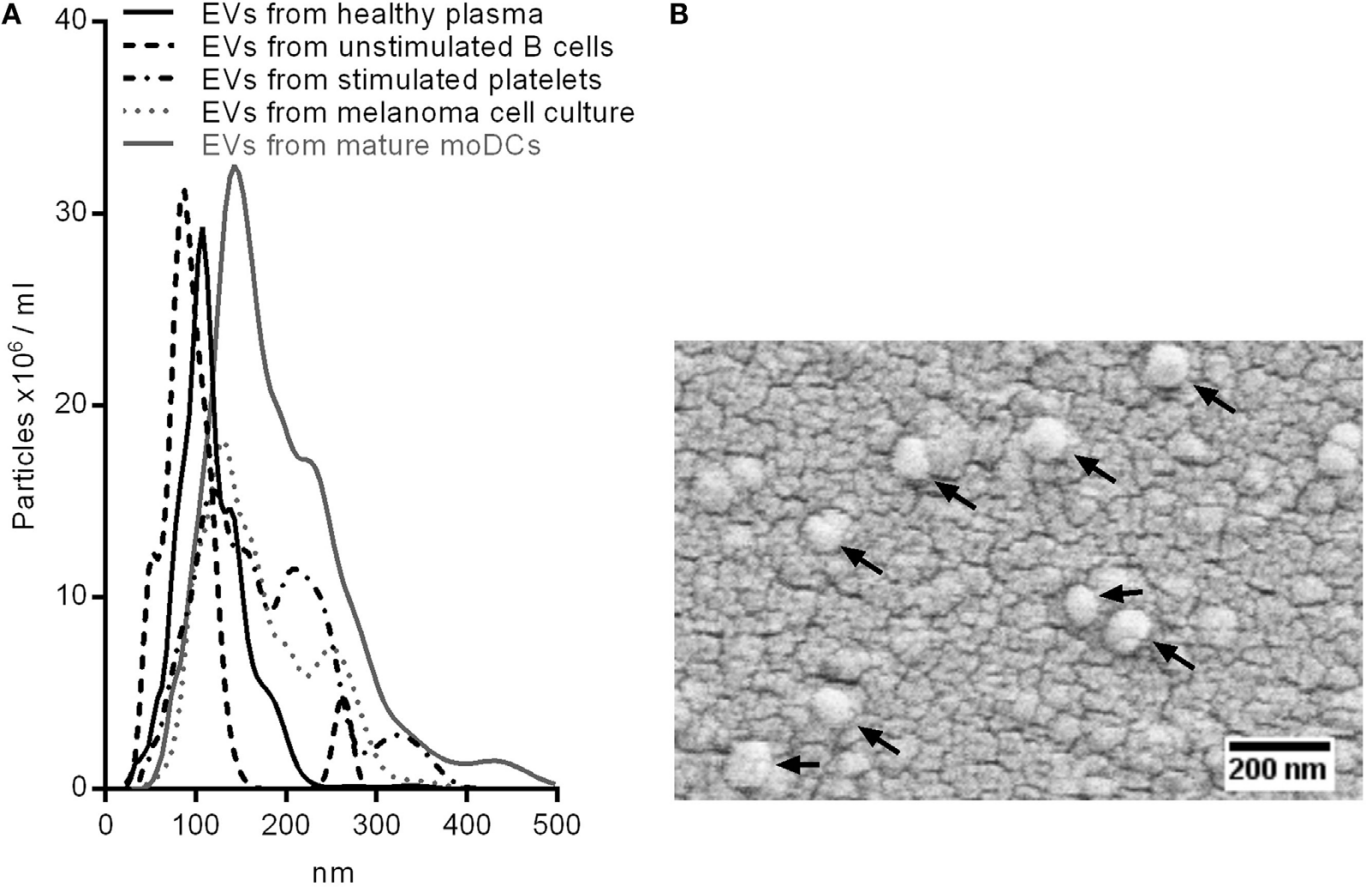
**Sizing of isolated EVs**. **(A)** Superimposed nanoparticle tracking analyses of EVs from healthy plasma, unstimulated B cells, Calcium Ionophore-stimulated platelets, melanoma cell culture, and mature monocyte-derived dendritic cells. **(B)** Scanning electron microscopy of EVs isolated from melanoma cell culture. Arrows point at EVs.

**Figure 2:**
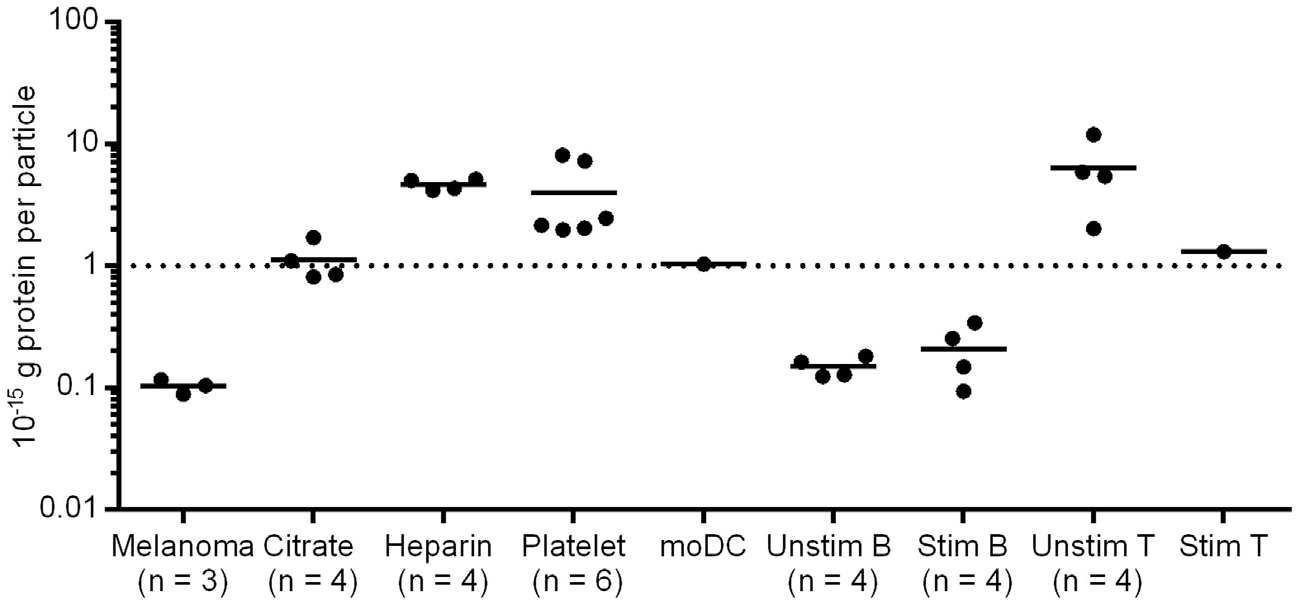
**Protein amount per particle of different EV samples**. Particle concentration was defined using nanoparticle tracking analysis (NTA); protein concentration was measured by BCA Assay. Melanoma EVs were isolated from primary melanoma cell cultures of three patients. Citrate/Heparin indicate the anticoagulant used for plasma preparation.

Extracellular vesicles from plasma of healthy donors or melanoma patients as well as from primary melanoma cell lines and isolated primary human blood cells were enriched for exosomes using ultracentrifugation. We determined the size of the isolated EVs by nanoparticle tracking analysis (NTA, Figure 1A), scanning electron microscopy (Figure 1B), and dynamic light scattering (Figure S1 in Supplementary Material). The peaks of the size distribution for the different EV types ranged from 90 to 140 nm (Figure 1A). NTA was also used to count particles and to calculate the protein amount per particle after protein quantification by BCA assay (Figure 2). Interestingly, we found the lowest protein amount per particle for EVs isolated from the melanoma cell culture supernatant. It was about 50 times lower as compared to most of the EV preparations from primary cells or plasma. The tumor cells might produce EVs with lower protein content or the other EV preparations might be contaminated with protein aggregates resulting in higher calculated protein amounts per particle.

Extracellular vesicles isolated from platelets, moDCs and T cells showed similar protein contents per particle as the plasma vesicles. By contrast, B cell EVs seemed to have a lower protein content. After B cell stimulation, EVs from resting and activated B cells did not differ significantly (*p* = 0.22, two-tailed paired *T*-test) in their protein amount (Figure 2).

### Surface Protein Profiles of Blood Cell-Derived EVs and Plasma EVs

Exosomes are known to incorporate proteins, including cell type-specific markers from their parental cell. To investigate the origin of EVs circulating in blood, the protein profiles of plasma EVs and blood cell-derived EVs were analyzed.

**Figure 3:**
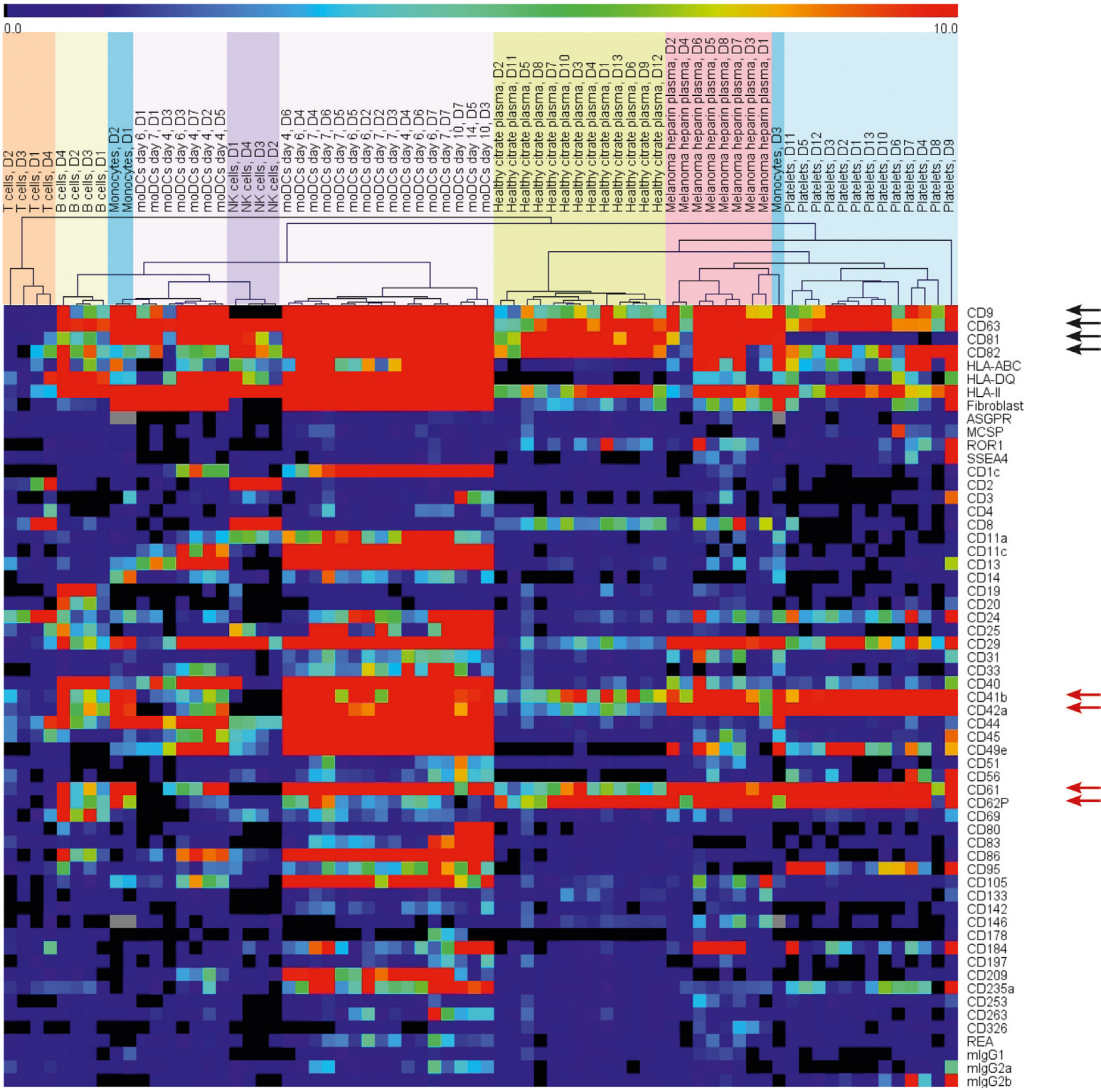
**Heat map depicting the surface protein profiles of EVs from different primary blood cells and plasma of healthy donors and melanoma patients**. Background corrected signals were used. The range indicator is limited to signals below 10 MFI to draw the attention to the presence or absence of signals instead of variations of strong signals.

The so-called exosome markers CD63 and CD82 were broadly detected, whereas CD9 and CD81 were present less frequently (Figure 3, black arrows). CD9 signals were remarkably low on NK cell-derived EVs and CD81 was absent on platelet EVs. T cell EVs showed only low signal intensities for most of the markers, including CD9 and CD81, most likely due to the low EV yield. Other surface proteins, such as CD29 (integrin beta1), CD24, HLA-ABC, and HLA class II were commonly detected.

Unsupervised hierarchical clustering grouped the EV samples according to their surface protein profiles and the originating cells (Figure 3). Within the selected surface proteins used for this assay, we detected cell-type-specific markers, such as CD2 and CD8 for T cell EVs, CD19 on B cell EVs, CD14 on monocyte EVs, or CD40, CD80, CD83, and CD86 on moDC-derived EVs. The platelet markers CD41b, CD42a, CD61, and CD62P were detected in most of the EV samples (Figure 3, red arrows) except from T cells and NK cells. T cell EVs showed generally low signal intensities and the NK cell EVs were isolated from NK cells after 14 days in culture with media changes. Due to the sticky nature of platelets, it is hard to completely avoid platelet contaminations if cells are isolated from buffy coats. Still, we preferred to use primary cells for the generation and analysis of EVs as EVs from cell lines might resemble an artificial phenotype. As a precaution, we would assign positive signals on platelet marker beads for non-platelet or plasma samples to platelet-derived EVs.

In the plasma samples, we observed some surface proteins corresponding to EVs from the cultured primary cells. We found the platelet markers CD41b, CD42a, CD61, and CD62P, the T cell or NK cell marker CD8, and finally CD40 and CD86 that implied the existence of dendritic cell EVs and B cell EVs. Three out of the 13 plasma vesicle preparations from healthy donors showed signals for CD19 that hint at B cell EVs in plasma. In summary, the surface proteins on plasma EVs indicated that mainly platelets, T cells, NK cells, dendritic cells, and B cells contribute to the pool of plasma EVs (Figure 3).

### The Composition of Surface Proteins on EVs Is Modified by Cell Stimulation

Extracellular vesicles circulating in blood might derive from resting as well as activated cells. The activation of some cells leads to an increased EV secretion and might also impact the protein composition of the secreted EVs. As an example, we isolated EVs from resting T cells and from stimulated T cells.

We observed stronger signals for each analyzed surface protein for EVs from stimulated T cells as compared to the unstimulated control samples. As the same protein amounts were used for the assay, the higher signals could be due to a lower proportion of potentially co-purified protein aggregates or due to an increased proportion of the detected surface proteins within the total protein amount. However, the signal increase was not statistically significant (data not shown).

**Figure 4:**
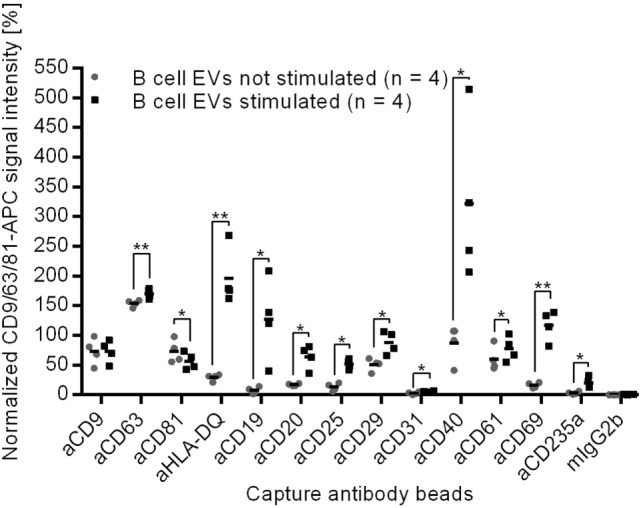
Normalized protein profiles of B cell-derived EVs without (gray dots) and after cell stimulation with CD40 ligand and IL-4 (black squares) for 4 days, *p < 0.05, **p < 0.01 (two-tailed paired *T*-test).

By contrast, EVs from stimulated B cells showed significantly altered signals for 12 surface proteins in comparison to EVs from unstimulated B cells (Figure 4). Eleven out of 12 significantly changed proteins showed a stronger signal, while the CD81 signal was weaker on EVs from stimulated B cells as compared to EVs from resting B cells. As the protein load per vesicle was comparable with and without B cell stimulation (Figure 2), the data indicate that the composition of the EVs was changed as a consequence of the stimulation.

Given that CD19, CD40, and CD69 are selectively loaded on EVs of stimulated B cells (Figure 4), the parallel detection of these surface proteins on plasma EVs from three donors (Figure 3) supports the presumption that some plasma vesicles might be secreted by activated B cells.

### The Composition of Surface Proteins on EVs Is Modified by moDC Differentiation and Maturation

As dendritic cells constitutively secrete EVs, plasma EVs might also derive from immature and mature dendritic cells. Therefore, EVs from monocytes, immature moDCs, and mature moDCs were analyzed.

**Figure 5:**
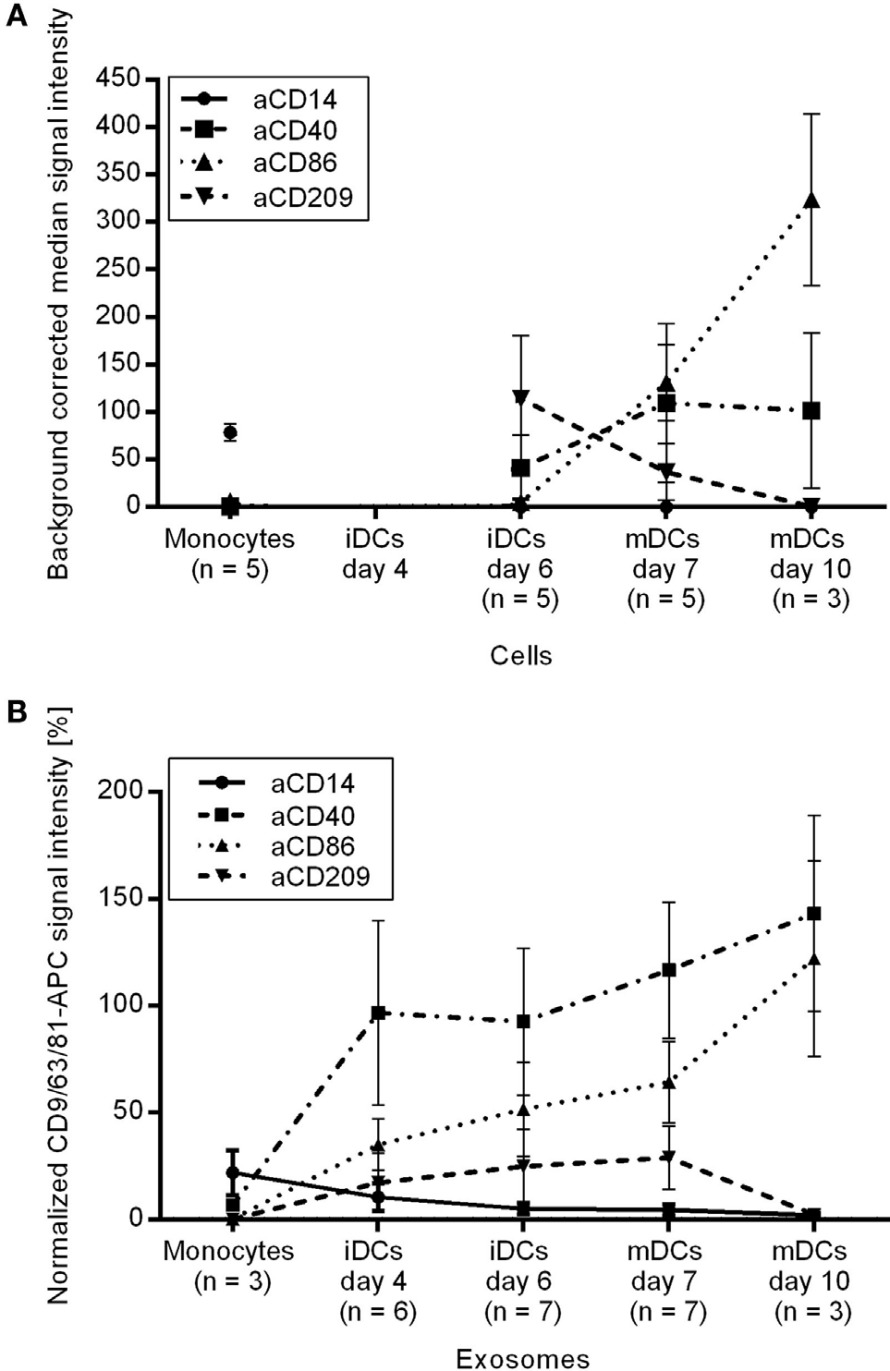
**Protein profiles of (A) monocytes, immature and mature moDCs after dead cell exclusion by PI and of (B) the respective EVs**.

The monocyte marker CD14 was detected on monocytes and their secreted vesicles while its signal intensity decreased during moDC differentiation on cells as well as on EVs (Figure 5). An opposing trend appeared for the activation markers CD40 and CD86 as the median signal intensities increased markedly on the moDCs during maturation and on the corresponding EVs (Figure 5), indicating that the activation markers are transferred from the cells to their EVs. The signals for CD209 (DC-SIGN) decreased during maturation on moDCs and later also on the respective EVs (Figure 5). In summary, activation markers known to be modulated during the generation of moDCs were also observed on the secreted EVs.

Plasma EVs from healthy donors showed weak signals for CD209 and CD86 and plasma EVs from five donors were positive for CD40. It should be considered with caution whether CD40^+^ EVs in plasma might derive from dendritic cells as they might also derive from activated B cells.

### Phenotyping of Plasma EVs from Melanoma Patients

We aimed to investigate whether the composition of EVs in the plasma of melanoma patients is different as compared to healthy controls and whether melanoma-specific EVs can be detected.

**Figure 6:**
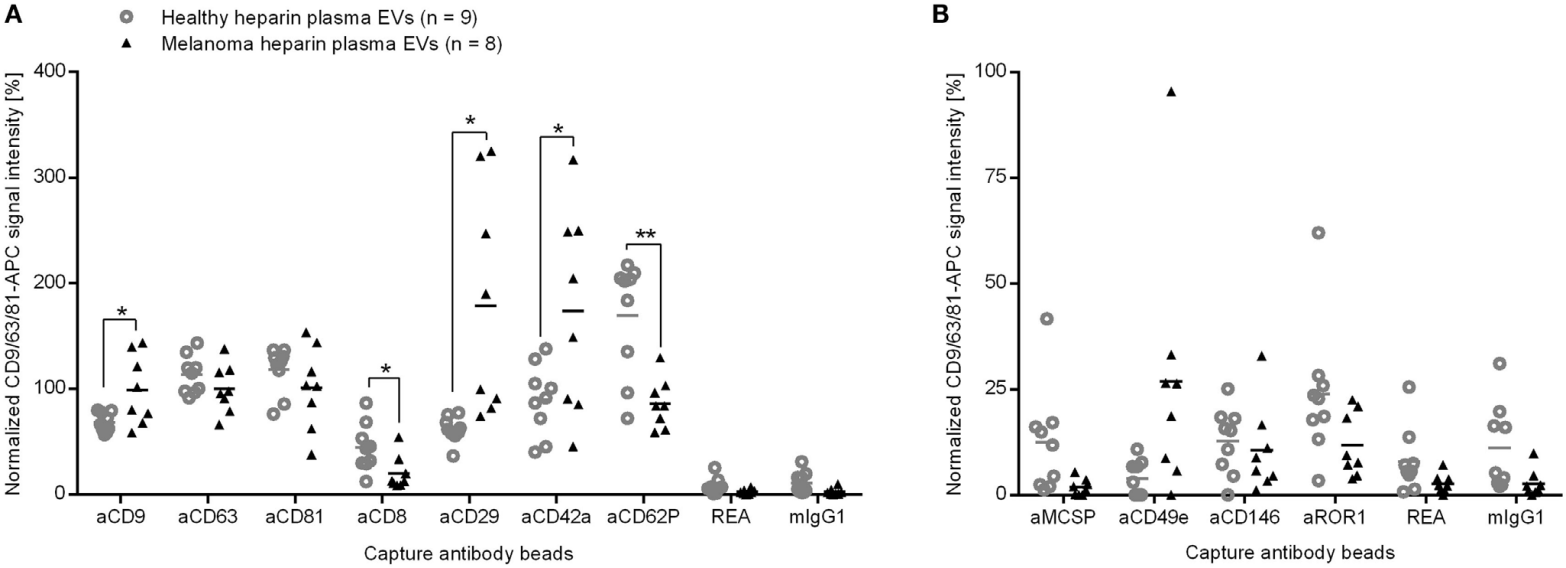
**Normalized signal intensities of (A) exosome markers and surface proteins that differ between plasma EVs of healthy controls or melanoma patients and (B) potential markers for melanoma EVs**. REA and mIgG1: isotype controls. *p < 0.05, **p < 0.01 (two-tailed *T*-test).

The plasma EVs of eight melanoma patients appeared distinct from heparin plasma EVs from nine healthy donors. Despite the use of different anticoagulants, the difference can also be seen between heparin melanoma plasma EVs and citrate plasma EVs from 13 healthy donors in the cluster analysis of surface proteins (Figure 3). To identify surface proteins on EVs that led to this separation, we calculated the fold change for each of 39 investigated surface proteins between heparin plasma EVs from healthy donors and melanoma patients. The signal intensities for five surface proteins differed by at least 1.3-fold, namely the general exosome marker CD9 as well as CD8, CD29, and the platelet markers CD42a and CD62P (Figure 6A). The signals for CD9, CD29, and CD42a were significantly increased (*p* < 0.05, two-tailed *T*-test, unequal variances; 1.4-fold, 2.9-fold, and 1.9-fold stronger, respectively). The signals for CD8 and CD62P were significantly decreased (*p* < 0.05; *p* < 0.01) and 2.2- and 2.0-fold lower, respectively, for EVs from melanoma patients as compared to healthy donors.

To exclude other variables than melanoma that may influence the surface protein signals for plasma EVs, we reanalyzed the data after splitting the samples of healthy donors by sex, age, and smoking habits. We saw only minor impacts on few EV protein signals (Figures S2, S3, and S4 in Supplementary Material). Additionally, we evaluated the impact of a potential delay between venipuncture and plasma isolation by preparing plasma from healthy donors with heparin as anticoagulant for different time points after blood draw and analyzed the isolated EVs. The calculation of correlation coefficients that involved 39 surface proteins showed that the time between blood draw and plasma isolation had hardly any effect (Table S1 in Supplementary Material). Two (CD29 and CD42a) out of the five proteins that showed signal differences between heparin plasma EVs from melanoma patients and healthy donors also showed changed signals between heparin and citrate plasma EVs from healthy donors (Figure 6A and Figure S4 in Supplementary Material). However, the average signal differences between healthy citrate plasma EVs and melanoma heparin plasma EVs for each of the five markers (CD9, CD8, CD29, CD42a, and CD62P) were at least equal or even more pronounced as compared to the differences of the heparin plasma samples (Figure S4 in Supplementary Material).

CD29 was detected on each EV preparation and can, therefore, not be attributed to a specific parental cell type. We detected CD42a on platelet EVs while CD81 was lacking (Figure 3) and, thus, considered an elevated proportion of platelet EVs in melanoma plasma. However, lower signals for the platelet marker CD62P on plasma EVs from melanoma patients might rather indicate a changed loading of platelet EVs than a higher proportion of platelet EVs. We detected CD8 on EVs from T cells and NK cells (Figure 3). Potentially, less EVs from cytotoxic cells, such as T cells and NK cells are the reason for the lower CD8 signals in melanoma plasma.

Surprisingly, we did not observe significant differences for the suggested melanoma markers melanoma-associated chondroitin sulfate proteoglycan (MSCP), CD146 (Mel-CAM), or CD49e (integrin α5) between plasma EVs from melanoma patients and healthy donors. All three markers were clearly detectable on melanoma EVs isolated from primary melanoma cell culture (data not shown). But only the CD49e signal appeared higher for plasma EVs from five of the eight melanoma patients (Figure 6B). By spike in experiments, we determined the sensitivity of the assay to be 1% for MCSP or CD146 of melanoma cell culture-derived EVs in plasma EV samples from healthy donors (Figure S5 in Supplementary Material). The higher detection limit of 10% for CD49e raises the question whether the CD49e signal in the plasma samples of melanoma patients could be addressed to tumor cells. As the integrin CD49e is not specific for tumor cells, the results should be interpreted with care.

As we did not see significant differences between the signal intensities for MCSP or CD146 on plasma EVs from melanoma patients as compared to healthy controls, we conclude that less than 1% of plasma EVs derive directly from melanoma tissue.

In summary, we observed that the signal intensities for five surface proteins differed significantly between plasma EVs from melanoma patients and healthy donors, but only a minor portion of plasma EVs derive from tumor cells.

## Discussion

Vesicles circulating in blood might derive from a variety of cells, including tumor cells in patients. It has been shown that immune cell-derived exosomes and tumor exosomes influence immune response and tumor progression (1, 22, 39, 40). Therefore, we focused our analysis on the potential contribution of EVs from immune cells and melanoma cells to the pool of EVs in plasma from melanoma patients.

As the so-called exosome markers CD9 and CD81 were missing on NK cell EVs or platelet EVs, respectively (32), we consider CD63 to be more suitable as general exosome marker. We detected CD63 signals also on plasma EVs in contrast to Jorgensen and colleagues (41) probably due to the use of an alternative antibody clone.

We detected the glycoproteins CD29 (integrin beta1) and CD24 on almost every EV preparation. CD29 is known as an interaction partner of tetraspanins on cells (42–44) and is probably transferred together with tetraspanins to exosomes during their biogenesis. Accordingly, CD29 had already been detected on different types of exosomes (45–48). CD24 is a GPI-anchored protein that is restricted to lipid rafts and is, therefore, likely also found on exosomes (49). According to our results, we suggest CD24 and CD29 along with CD63 as common exosome proteins that are applicable as staining markers for EVs.

Major histocompatibility complex class I (HLA-ABC) is expressed by practically every nucleated cell and has been shown to be transferred to exosomes (1). The low signal intensities for HLA-ABC on plasma EVs might be due to a high percentage of MHC class I weak EVs, such as those secreted by NK cells and platelets.

Besides the broadly detected surface proteins on EVs, more specific markers were identified that allow to determine cell types potentially contributing to the pool of plasma EVs. We detected platelet markers (CD41b, CD42a, CD61, CD62P) on plasma vesicles in line with previous studies (17, 50), confirming the assumption that platelets contribute to the pool of plasma EVs. APCs are considered as another source of plasma EVs because HLA-DR positive signals had been described for plasma EVs captured on anti-CD63-coated beads (17, 18). However, the contribution of APCs might have been overestimated because we detected HLA class II also on platelet EVs. Therefore, the signals for CD40 and CD86, which were detected on moDC-EVs and B cell EVs, more likely reflect APC-EVs in plasma. Additionally, we detected signals for CD8 on plasma EVs, implying a contribution of T cells and NK cells to the mixture of vesicles in plasma.

Plasma EVs might also give information about cell–cell interactions, such as stimulation of immune cells. For example, T cell receptor activation had already been shown to upregulate the production of exosomes (20, 21). Despite the increased exosome release, we did not observe a significantly altered protein composition of EVs that could be used to distinguish EVs from resting T cells and stimulated T cells. By contrast, we observed that the signals of 12 surface proteins were changed on EVs if B cells were stimulated. Among the 12 markers, exosomal HLA has already been reported to be increased after B cell stimulation (19, 51). CD20 is known to be downregulated on B cells upon CD40 activation (52) and has been detected on B cell exosomes (26, 27). After B cell activation, CD20 as a B cell co-receptor (53), CD40 as a co-stimulatory molecule, and CD69 as a known activation marker (54) might be transferred via exosomes to resting B cells to enhance immune responses.

To analyze EVs of dendritic cells, we used moDCs due to the low frequency of dendritic cells in blood, even though it is controversial whether *in vitro* generated moDCs are comparable with dendritic cells in blood (55, 56). The signals for HLA class II and CD40 on plasma EVs could be an indication of DCs or activated B cells.

Comparing EVs from plasma of melanoma patients and healthy controls, we found that the signals of 5 out of 39 investigated proteins differed significantly and by at least 30%, namely CD9, CD8, CD29, CD42a, and CD62P. The platelet markers CD42a and CD62p indicate that either the proportion of platelet EVs or the loading of platelet EVs is altered in melanoma patients. Platelet microparticles were shown to induce metastasis and angiogenesis in lung cancer (57) and invasiveness in breast cancer (58). A surplus of platelet EVs might, therefore, indicate tumor progression.

The lower signal for CD8 on melanoma plasma EVs as compared to healthy plasma EVs could be due to less NK or T cell EVs. NK cell-derived exosomes are known to carry Fas ligands that mediate tumor cell killing (59) and T cell-derived EVs are thought to transfer functional miRNAs to APCs to generate an immune response (60). EVs from NK cells and T cells are, thus, able to support immune responses against tumors. We hypothesize that, along with the previously discussed elevated proportion of platelet–EVs, a downsized fraction of EVs from cytotoxic cells might indicate tumor progression and an attenuated immune response.

We analyzed and tried to exclude technical reasons for the altered composition of plasma EVs in melanoma patients. In line with Yuana and colleagues, we did not observe increasing signals for platelet markers on plasma EVs with time after sample preparation (61).

As the melanoma EV samples were isolated from heparin plasma, we compared the protein profiles with the protein profiles of healthy control EVs prepared also from heparin plasma. However, heparin is not able to chelate calcium ions and still allows platelet degranulation that leads to EV secretion. Accordingly, citrate is recommended in EV research as anticoagulant to prevent EV release after blood draw (62, 63). To investigate the impact of the anticoagulant on the protein profile of plasma EVs, we also included EVs from healthy plasma anticoagulated with citrate in our analysis. Differing signals for CD29, and CD42a between heparin and citrate plasma EVs from healthy donors implied an anticoagulant-mediated effect. However, the differences for CD29 and CD42a appeared even stronger if healthy citrate plasma EV samples were compared to the heparin plasma samples of melanoma patients.

Tumor-derived EVs in plasma are discussed as potential biomarkers because they were described as “relatively numerous” and easily accessible (64). We could hardly detect signals for the potential melanoma markers MSCP or CD146 (Mel-CAM) on EVs from plasma of melanoma patients. We also considered CD49e (integrin α5) as a potential marker for melanoma-derived EVs because it was found to be expressed by melanoma cells (33) and we detected CD49e signals on EVs from melanoma cell cultures. On plasma EVs, the CD49e signals were increased for five out of eight melanoma patients.

However, in EVs isolated from melanoma cell culture, the CD49e signal was less pronounced as compared to MSCP or CD146. Therefore, we consider the CD49e signal to be related to EVs from non-tumor cells because integrins are not tumor specific. We conclude that melanoma EVs are not a major portion within the pool of EVs in melanoma patient’s plasma.

For our multiplex platform, we determined a detection limit of 1% melanoma EVs in plasma. To estimate the tumor size required to secrete EVs giving at least 1% of plasma EVs, we considered that we isolated on average 7 μg EVs per milliliter whole blood from healthy donors. Approximately 80 μg EVs were secreted from 10^9^ melanoma cells in culture. Supposing 5 liter blood per person and the volume of a melanocyte to be in the range of 600 μm^3^ (65), we calculated that a well vascularized melanoma of at least 2600 mm^3^ (e.g., diameter of 28 mm and 4 mm depth) in size could give rise to EV numbers that are equivalent to 1% of plasma vesicles. Even for stage 3 or 4 patients, the requested tumor size would be quite big or many metastasis would be needed to sum up to the calculated number of tumor cells. Due to lacking signals for the potential melanoma markers MCSP or CD146 on plasma EVs from melanoma patients, we conclude that there are less than 1% melanoma EVs circulating in these patient’s plasma. Interestingly, Laresche and colleagues also were unable to detect melanoma microparticles in the plasma of over 100 melanoma patients (66).

Taken together, our data indicate that the majority of plasma EVs of melanoma patients do not derive directly from the tumor. However, differing marker signals for platelets and cytotoxic cells on plasma EVs from melanoma patients and healthy donors point at an altered proportion or marker loading of EVs. Potentially, this variation of plasma EVs is related to enhanced vascularization or diminished anti-tumor immune responses. Although we investigated only eight patient samples, we observed significant differences in plasma EV protein profiles. It will be interesting to analyze the plasma EVs of more melanoma patients with different tumor stages to investigate whether the composition and protein loading of plasma EVs reflect the influence of the immune system on tumor progression.

## Author Contributions

NK isolated primary cells and extracellular vesicles, performed dynamic light scattering, established the multiplex bead-based platform, characterized isolated cells and extracellular vesicle by flow cytometry, analyzed the data, and drafted the manuscript. UH isolated natural killer cell-derived exosomes and established the multiplex bead-based platform. TO isolated monocytes for differentiation and maturation into monocyte-derived dendritic cells, analyzed the cells by flow cytometry, and revised the manuscript. MW performed nanoparticle tracking analysis and revised the manuscript. AB analyzed the data and contributed in writing the manuscript. SW designed the study, analyzed the data, and contributed in writing the manuscript. All authors read and approved the final manuscript.

## Conflict of Interest Statement

NK, UH, TO, AB, and SW are employed at Miltenyi Biotec GmbH. A patent application has been submitted for the multiplex bead platform: A method for analyzing markers on the surface of vesicles, submission number 3496895, application number EP15167673.1.

## Abbreviations

APC(s), antigen-presenting cell(s); BCA assay, bicinchoninic acid assay; BSA, bovine serum albumin; CCR7, C–C chemokine receptor type 7; CD, cluster of differentiation; CXCR4, C–X–C chemokine receptor type 4; D, donor; DC, dendritic cell; EDTA, ethylenediaminetetraacetate; ELISA, enzyme-linked immunosorbent assay; EV(s), extracellular vesicle(s); GM-CSF, granulocyte-macrophage colony-stimulating factor; GPI, glycosylphosphatidylinositol; HLA(-ABC, DP, DQ, DR), human leukocyte antigen (type ABC, DP, DQ, DR); ICAM-1, intercellular adhesion molecule 1; iDC(s), immature dendritic cells(s); IL, interleukin; MCSP, melanoma-associated chondroitin sulfate proteoglycan; Mel-CAM, melanoma cell adhesion molecule; MHC, major histocompatibility complex; moDC(s), monocyte-derived dendritic cell(s); NK cell(s), natural killer cell(s); NTA, nanoparticle tracking analysis; PAP, PBS supplemented with 0.1% pluronic and 0.09% azide; PGE, prostaglandin; ROR1, tyrosine protein kinase transmembrane receptor.
