# Supplementary material for: Melanoma Affects the Composition of Blood Cell-Derived Extracellular Vesicles
Source: Front Immunol. 2016 Jul 26;7:282. doi: 10.3389/fimmu.2016.00282 (PMC4960424; doi:10.3389/fimmu.2016.00282)
Supplement: Supplementary file 1 [file Table_1.pdf]

Supplementary Material

Melanoma affects the composition of blood cell-derived extracellular vesicles

Nina Koliha, Ute Heider, Tobias Ozimkowski, Martin Wiemann, Andreas Bosio, Stefan Wild*

*** Correspondence:** Stefan Wild, Stefan.Wild@miltenyibiotec.de

Supplementary results and discussion

To verify that the surface markers on plasma EVs are changed due to melanoma and not because of other variables, we investigated the impact of the anticoagulant and the time between venipuncture and plasma isolation. We suspected these variables to potentially impact the composition of plasma EVs because the blood from melanoma patients was drawn for monitoring analyses, anticoagulated with heparin and might be processed with delays. But citrate was used for the blood of healthy donors as it is recommended in EV research (Mullier et al., 2013; Witwer et al., 2013) and the blood samples were processed directly. A correlation plot with 39 EV surface markers demonstrated that melanoma has a more dramatic impact on the composition of plasma EVs than the anticoagulant or the potential delay during sample preparation (Supplementary table 1).

Some of the markers that we identified as changed due to melanoma are also changed between plasma EVs from blood containing heparin or citrate, namely CD29 and CD42a. However, the difference of these markers between plasma EVs from healthy donors with citrate and plasma EVs from melanoma patients with heparin appear even more pronounced (Supplementary figure 4).

Supplementary figures and tables

Melanoma EVs

Healthy plasma EVs

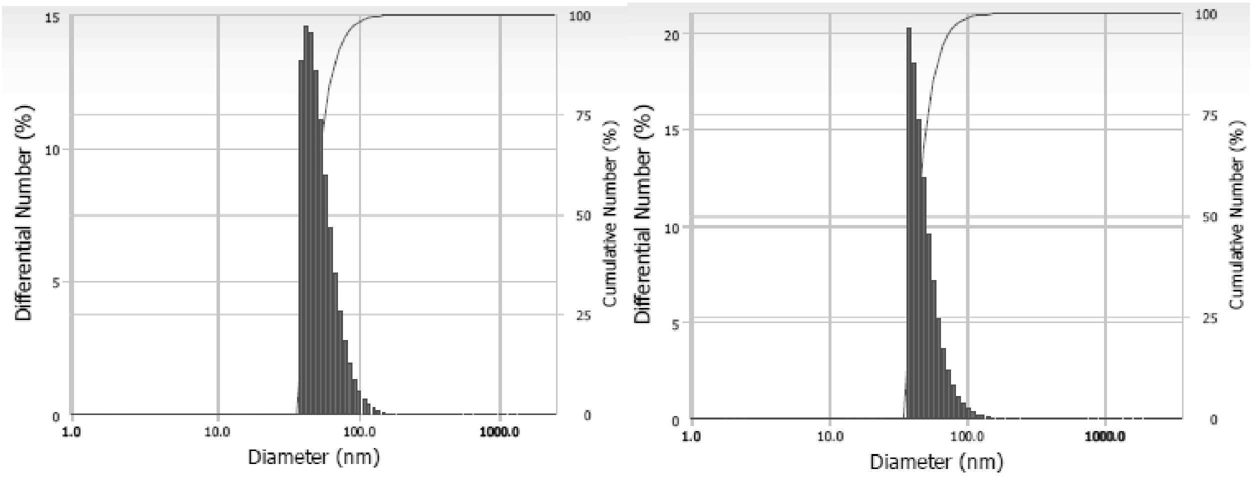

Supplementary figure 1: Sizing of EVs using dynamic light scattering (DLS). 98% of EVs were smaller than 100 nm.

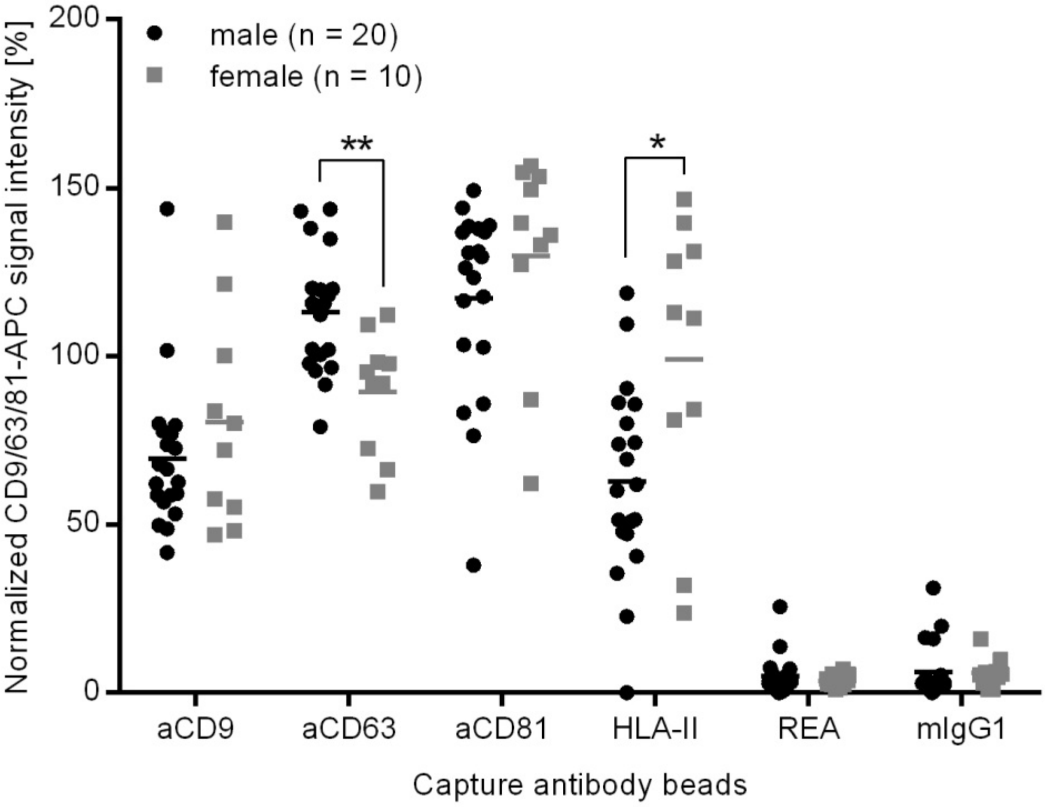

Supplementary figure 2: Normalized signal intensities of surface proteins that differed significantly between plasma EVs from male and female donors. The signals for CD9 and CD81 are comparable between the two groups and are depicted for comparison. REA and mIgG1: isotype controls.

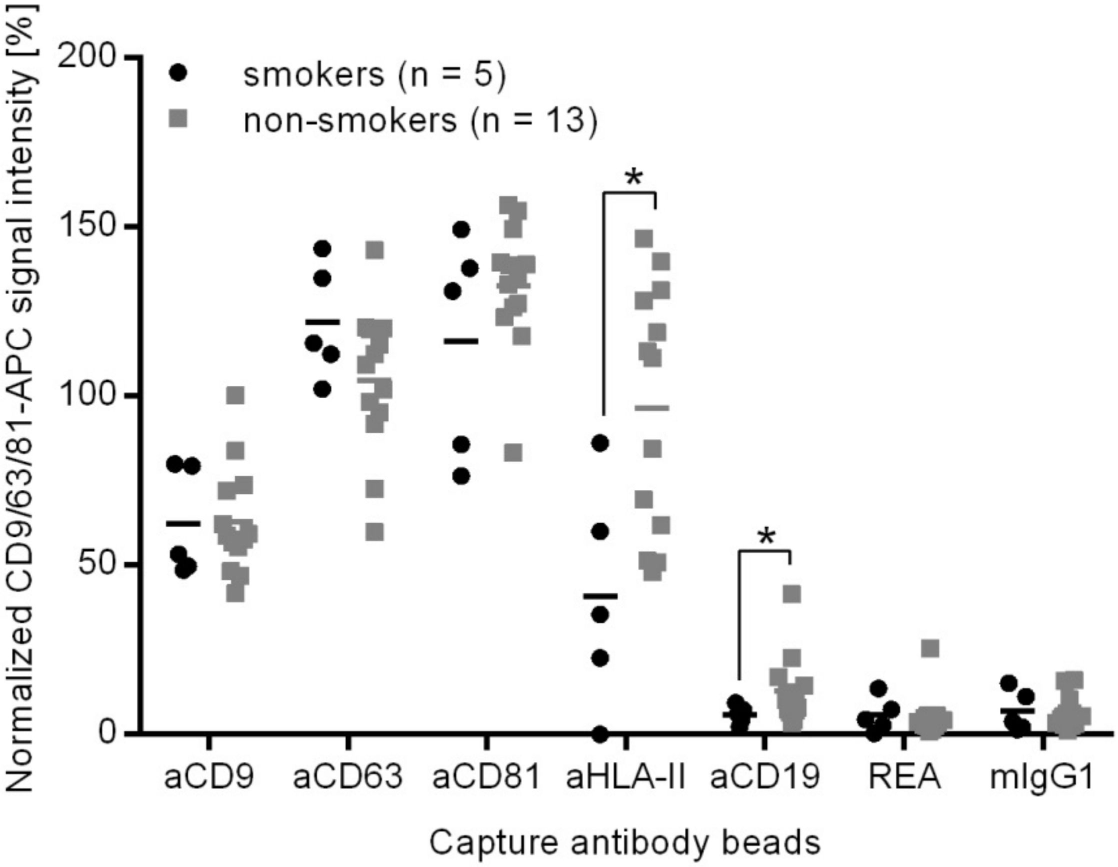

Supplementary figure 3: Normalized signal intensities of surface proteins that differed significantly between plasma EVs from healthy donors that are smokers or non-smokers. The signals for CD9, CD63 and CD81 are comparable between the two groups and are depicted for. REA and mIgG1: isotype controls.

Supplementary table 1: Correlation plot of normalized signal intensities of 39 surface proteins that were averaged for the investigated samples, including plasma EVs from blood of healthy donors with citrate or heparin, plasma EVs from blood of melanoma patients with heparin and plasma EVs from healthy donors with heparin and no delay (0 min), 30 min delay or 120 min delay between blood draw and plasma isolation.

	Citrate	Heparin	Melanoma	0 min	30 min	2 h
Citrate		0.93	0.61	0.93	0.94	0.95
Heparin	0.93		0.80	0.96	0.96	0.95
Melanoma	0.61	0.80		0.74	0.69	0.65
0 min	0.93	0.96	0.74		0.99	0.98
30 min	0.94	0.96	0.69	0.99		0.99
2 h	0.95	0.95	0.65	0.98	0.99	

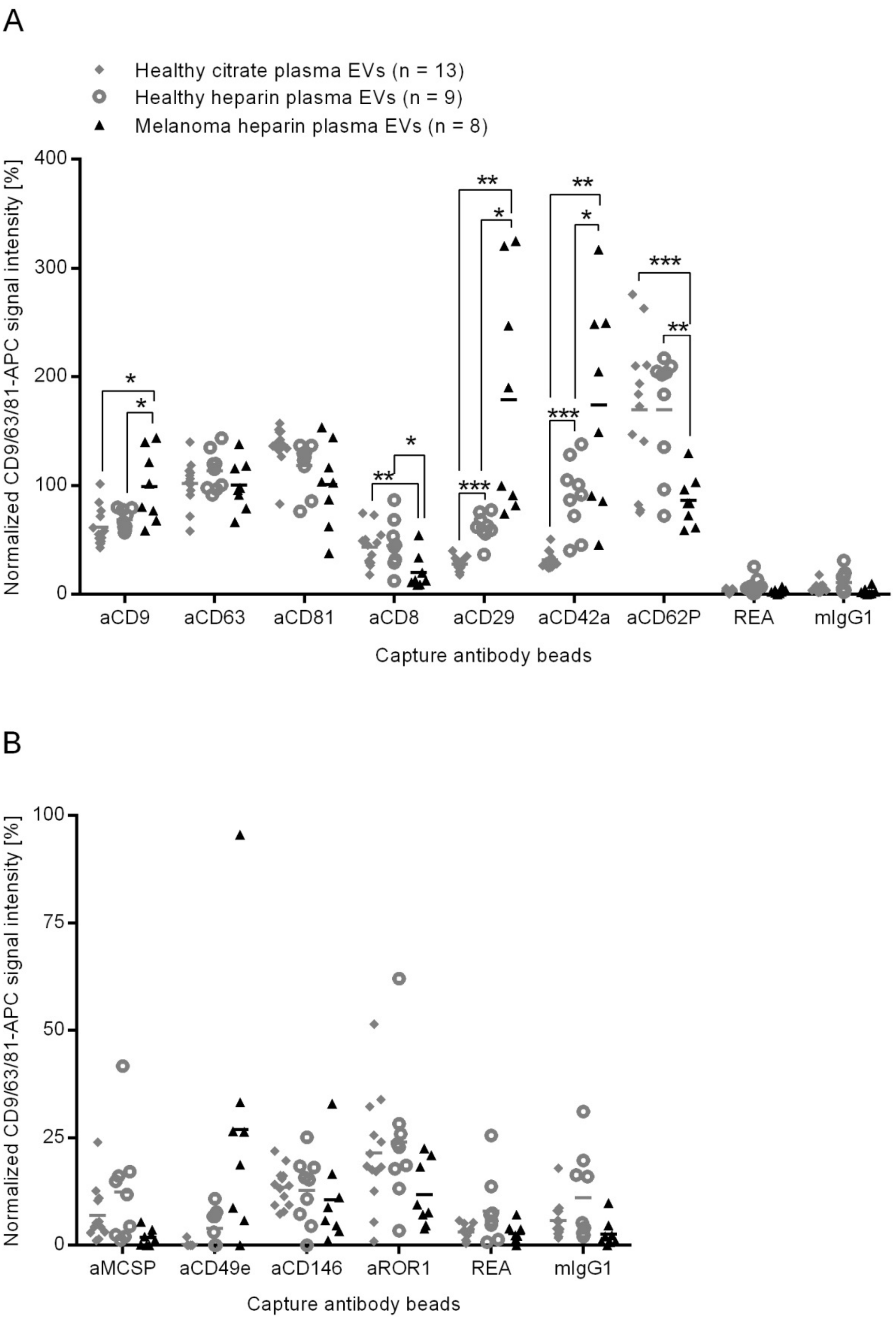

Supplementary figure 4: Normalized signal intensities of (**A**) exosome markers and proteins that differed significantly between plasma EVs of melanoma patients and healthy donors and (**B**) potential melanoma markers. REA and mIgG1: isotype controls.

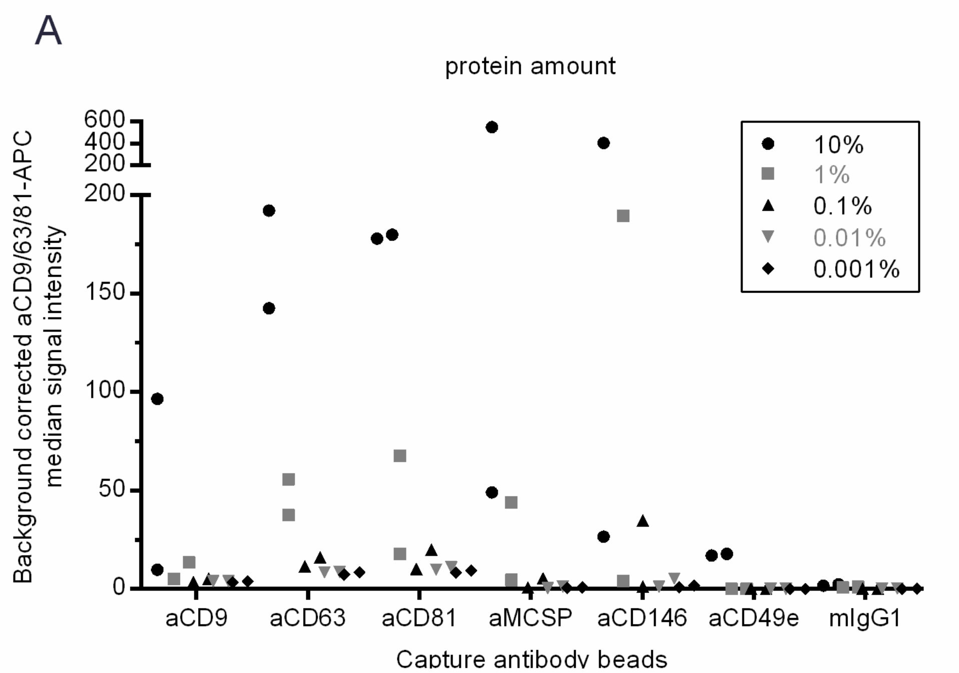

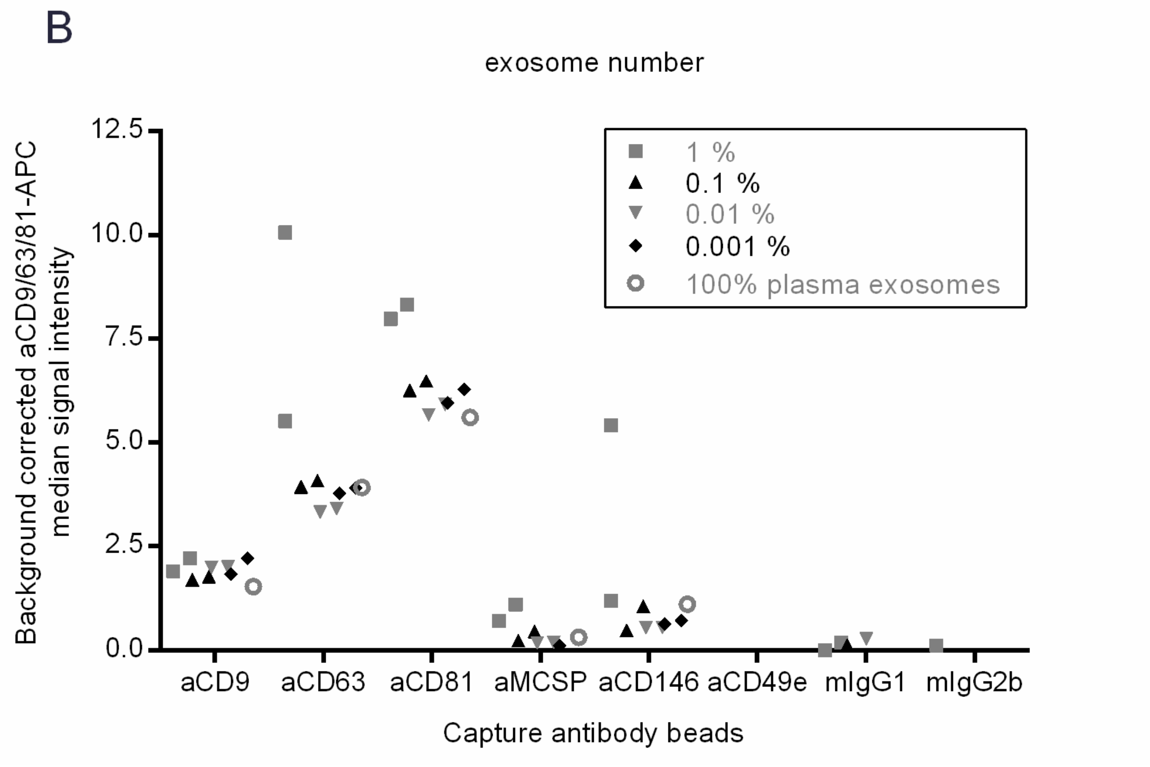

Supplementary figure 5: Spike-in of melanoma EVs from two different primary melanoma cell cultures into a plasma EV sample from a healthy donor. (**A**) The percentage of melanoma EVs refers to the total protein amount in microgram measured by BCA assay, 1% are 0.64 µg or ~$60x10^8$ particles. (**B**) The percentage of melanoma EVs refers to the total number of particles measured by NTA, 1% are 0.02 µg or ~$1.8x10^8$ particles. mIgG1: isotype control.

Supplementary table 2: Overview of healthy donors and melanoma patients. N.a. = not applicable.

<table>
<tr><th></th><th>Donor no</th><th>Age in 2015</th><th>sex</th><th>Smoking habit</th><th>Medication</th><th>Needle type, tube type</th></tr>
<tr><td rowspan="13">Healthy donors with citrate</td><td>D1</td><td>29</td><td>f</td><td>non-smoker</td><td>Hormonal contra-ception</td><td rowspan="13">Safety Blood Collection Set, Greiner, 21G x ¾”, 0.105M sodium citrate Vacutainer, BD</td></tr>
<tr><td>D2</td><td>42</td><td>f</td><td>non-smoker</td><td>Thyronaiod 50</td></tr>
<tr><td>D3</td><td>41</td><td>m</td><td>non-smoker</td><td>non</td></tr>
<tr><td>D4</td><td>33</td><td>f</td><td>non-smoker</td><td>non</td></tr>
<tr><td>D5</td><td>33</td><td>f</td><td>non-smoker</td><td>non</td></tr>
<tr><td>D6</td><td>34</td><td>f</td><td>non-smoker</td><td>non</td></tr>
<tr><td>D7</td><td>24</td><td>m</td><td>smoker</td><td>non</td></tr>
<tr><td>D8</td><td>44</td><td>m</td><td>smoker</td><td>non</td></tr>
<tr><td>D9</td><td>46</td><td>f</td><td>non-smoker</td><td>non</td></tr>
<tr><td>D10</td><td>44</td><td>m</td><td>non-smoker</td><td>Berodual if needed</td></tr>
<tr><td>D11</td><td>37</td><td>f</td><td>non-smoker</td><td>non</td></tr>
<tr><td>D12</td><td>33</td><td>m</td><td>smoker</td><td>non</td></tr>
<tr><td>D13</td><td>37</td><td>m</td><td>non-smoker</td><td>non</td></tr>
<tr><td rowspan="9">Healthy donors with heparin</td><td>D1</td><td>40</td><td>m</td><td>non-smoker</td><td>n. a.</td><td rowspan="9">Safety Blood Collection Set, Greiner, 21G x ¾”, 17 IU/ml heparin, BD</td></tr>
<tr><td>D2</td><td>33</td><td>m</td><td>smoker</td><td>n. a.</td></tr>
<tr><td>D3</td><td>24</td><td>m</td><td>smoker</td><td>n. a.</td></tr>
<tr><td>D4</td><td>37</td><td>m</td><td>non-smoker</td><td>n. a.</td></tr>
<tr><td>D5</td><td>37</td><td>m</td><td>non-smoker</td><td>n. a.</td></tr>
<tr><td>D6</td><td>32</td><td>m</td><td>n. a.</td><td>n. a.</td></tr>
<tr><td>D7</td><td>63</td><td>m</td><td>n. a.</td><td>n. a.</td></tr>
<tr><td>D8</td><td>38</td><td>m</td><td>n. a.</td><td>n. a.</td></tr>
<tr><td>D9</td><td>25</td><td>m</td><td>n. a.</td><td>n. a.</td></tr>
<tr><td rowspan="8">Melanoma patients</td><td>D1</td><td>70</td><td>m</td><td>n. a.</td><td>n. a.</td><td></td></tr>
<tr><td>D2</td><td>75</td><td>f</td><td>n. a.</td><td>n. a.</td><td></td></tr>
<tr><td>D3</td><td>46</td><td>m</td><td>n. a.</td><td>n. a.</td><td></td></tr>
<tr><td>D4</td><td>48</td><td>m</td><td>n. a.</td><td>n. a.</td><td></td></tr>
<tr><td>D5</td><td>50</td><td>f</td><td>n. a.</td><td>n. a.</td><td></td></tr>
<tr><td>D6</td><td>53</td><td>m</td><td>n. a.</td><td>n. a.</td><td></td></tr>
<tr><td>D7</td><td>75</td><td>m</td><td>n. a.</td><td>n. a.</td><td></td></tr>
<tr><td>D8</td><td>53</td><td>f</td><td>n. a.</td><td>n. a.</td><td></td></tr>
</table>

Supplementary table 3: List of antibodies used as capture antibodies bound to the polystyrene particles in the multiplex platform

	Capture antibody	Clone	Target	Supplier
1	Anti-ASGPR	8D7	Asialoglycoprotein receptor 1, liver cells	BD Biosciences
2	Anti-Fibroblast	REA165	fibroblasts, epithelial cells, peripheral blood myeloid cells, subset of lymphocytes	Miltenyi Biotec
3	CD9	SN4	Exosome marker	Miltenyi Biotec
4	CD63	H5C6	Exosome marker	Miltenyi Biotec
5	CD81	5A6	Exosome marker	BioLegend
6	CD82	REA	Exosome marker	Miltenyi Biotec
7	CD1c	AD5-8E7	BDCA-1, major subpopulation of human myeloid dendritic cells	Miltenyi Biotec
8	CD2	LT2.2	T cells, subset of NK cells	Miltenyi Biotec
9	CD3	BW 264/56	mature human T cells, thymocytes, subset of NK cells	Miltenyi Biotec
10	CD4	Vit-4.3	T helper cells, thymocytes, monocytes, dendritic cells	Miltenyi Biotec
11	CD8	BW 135/80	cytotoxic T cells, thymocytes, subset of NK cells	Miltenyi Biotec
12	CD11a	REA378	integrin α-L (ITGAL), leukocytes, T cells, granulocytes, monocytes, other	Miltenyi Biotec
13	CD11c	MJ4-27G12	integrin αX or CR4, monocytes, macrophages, NK cells, granulocytes, myeloid dendritic cells (MDCs), subsets of T and B cells	Miltenyi Biotec
14	CD13	REA263	granulocytes, myeloid progenitors, endothelial cells, epithelial cells, and subset of granular lymphoid cells	Miltenyi Biotec
15	CD14	TÜK4	monocytes and macrophages, subset of neutrophils and myeloid dendritic cells	Miltenyi Biotec
16	CD19	LT-19	B cells	Miltenyi Biotec
17	CD20	LT20.34	B lineage cells from the pre-B cell stage to the B cell lymphoblast stage	Miltenyi Biotec
18	CD24	32D12	heat-stable antigen (HSA)	Miltenyi Biotec
19	CD25	3G10	activated T and B cells, macrophages, subset of non-activated CD4+ regulatory T cells	Miltenyi Biotec
20	CD29	TS2/16.2.1	integrin beta 1	Miltenyi Biotec

	Capture antibody	Clone	Target	Supplier
21	CD31	AC128	monocytes, platelets, and granulocytes	Miltenyi Biotec
22	CD33	AC104.3E3	Monocytes, granulocytes, some myeloid dendritic cells	Miltenyi Biotec
23	CD40	HB14	B cells, macrophages, dendritic cells, endothelial cells, fibroblasts, plasma cells, subset of peripheral T cells	Miltenyi Biotec
24	CD41b	REA336	β chain of Integrin α-IIb, megakaryocytes, platelets	Miltenyi Biotec
25	CD42a	REA209	Platelets, megakaryocytes	Miltenyi Biotec
26	CD44	DB105	Cancer stem cells (CSC), hematopoietic, fibroblastic, and glial cells.	Miltenyi Biotec
27	CD45	5B1	leukocyte common antigen	Miltenyi Biotec
28	CD49e	NKI-SAM1	integrin α5 chain, lymphocytes, monocytes, fibroblasts, endothelial cells	Nordic-MUbio
29	CD51	REA181	αV integrin	Miltenyi Biotec
30	CD56	REA196	neural cell adhesion molecule (NCAM), resting and activated NK cells, minor subset of CD3+ T cells	Miltenyi Biotec
31	CD61	VI-PL2	integrin β3, megakaryocytes and platelets	BD Biosciences
32	CD62P	REA389	P-selectin, vascular endothelial cells and platelets	Miltenyi Biotec
33	CD69	FN50	Activated lymphocytes, monocytes, and platelets	Miltenyi Biotec
34	CD80	2D10	B7-1, activated B cells, dendritic cells, and monocytes/ macrophages	Miltenyi Biotec
35	CD83	HB15	activated dendritic cells	Miltenyi Biotec
36	CD86	FM95	B7-2, activated B and T cells, dendritic cells, and monocytes/macrophages	Miltenyi Biotec
37	CD95	DX2	Fas, Apo-1, activated lymphocytes	Miltenyi Biotec
38	CD105	43A4E1.71	mature endothelial cells, some leukemic cells of B lymphoid and myeloid origin	Miltenyi Biotec
39	CD133	AC133.1.6. 2.1.1	multipotent progenitor cells, including immature hematopoietic stem and progenitor cells, circulating endothelial progenitor cells, fetal neural stem cells, other tissue-specific stem cells, cancer stem cells	Miltenyi Biotec

	Capture antibody	Clone	Target	Supplier
40	CD142	HTF-1	Tissue factor, activated endothelial cells, monocytes, macrophages, platelets, and some tumor cell types	Miltenyi Biotec
41	CD146	541-10B2	MUC18, MCAM, Mel-CAM, endothelial cells, pericytes, smooth muscle cells, follicular dendritic cells, melanoma cells, sub-population of activated T lymphocytes, marrow stromal cells (MSCs)	Miltenyi Biotec
42	CD184	12G5	CXCR4, blood and tissue cells	Miltenyi Biotec
43	CD197	FR11-11E8.17.3	CCR7, B cells, dendritic cells, macrophages, NK cells, T cells, thymocytes	Miltenyi Biotec
44	CD209	DCN-47.5.4	DC-SIGN, dendritic cells, endothelial cells, macrophages, spleen	Miltenyi Biotec
45	CD235a	REA175	Glycophorin-A, mature erythrocytes and erythroid precursor cells	Miltenyi Biotec
46	CD253	RIK-2.1	TRAIL ligand, activated lymphocytes	Miltenyi Biotec
47	CD263	DJR3	TRAIL receptor, peripheral blood lymphocytes, spleen, skeletal muscle, placenta, lung and heart	Miltenyi Biotec
48	CD326	HEA125	EpCAM, basolateral surface of carcinoma and epithelial cells in tissues, circulating tumor cells, cancer stem cells, not on melanoma, neuroblastoma, sarcoma, lymphoma, leukemia cells, or normal fibroblasts	Miltenyi Biotec
49	HLA-ABC	REA230	Nuclear cells	Miltenyi Biotec
50	HLA-DQ	REA	Antigen-presenting cells	Miltenyi Biotec
51	HLA-DP/DQ/DR	REA332	Antigen-presenting cells	Miltenyi Biotec
52	MCSP	EP-1	melanoma-associated chondroitin sulfate proteoglycan antigen, melanoma tissues and melanoma cell lines but not carcinoma cells, fibroblastoid cells, and cells of hematopoietic origin	Miltenyi Biotec
53	ROR1	2A2	receptor tyrosine kinase-like orphan receptor 1, chronic lymphocytic leukemia (CLL) and mantle cell lymphoma (McLellan), ovarian cancer, renal cancer, melanoma, and lung adenocarcinoma, adipose tissue, at early stages of B cell development	Miltenyi Biotec

	Capture antibody	Clone	Target	Supplier
54	SSEA-4		Stage-specific embryonic antigen 4, undifferentiated human embryonic stem (ES) cells, induced pluripotent (iPS) cells embryonal carcinoma (EC) cells, and embryonic germ (EG) cells, somatic stem cells	Miltenyi Biotec
55	mIgG1	IS5-21F5	Isotype control	Miltenyi Biotec
56	mIgG2a	S43.10	Isotype control	Miltenyi Biotec
57	mIgG2b	IS6-11E5	Isotype control	Miltenyi Biotec
58	REA	REA293	Isotype control	Miltenyi Biotec

McLellan, A.D. (2009). Exosome release by primary B cells. *Critical reviews in immunology* 29, 203-217. doi:

Mullier, F., N. Bailly, C. Chatelain, B. Chatelain, and J.M. Dogne (2013). Pre-analytical issues in the measurement of circulating microparticles: current recommendations and pending questions. *Journal of thrombosis and haemostasis : JTH* 11, 693-696. doi: 10.1111/jth.12171

Witwer, K.W., E.I. Buzas, L.T. Bemis, A. Bora, C. Lasser, J. Lotvall, E.N. Nolte-'t Hoen, M.G. Piper, S. Sivaraman, J. Skog, C. Thery, M.H. Wauben, and F. Hochberg (2013). Standardization of sample collection, isolation and analysis methods in extracellular vesicle research. *Journal of extracellular vesicles* 2. doi: 10.3402/jev.v2i0.20360
